# Subcritical Water Extract from Grape Pomace Protects Human Bronchial Epithelium Cells by Mitigating Oxidative Stress Through Nrf2 Pathway

**DOI:** 10.3390/molecules31101736

**Published:** 2026-05-19

**Authors:** Federica Affranchi, Giovanni Pratelli, Raffaele Raimondo, Pavel Kiselev, Michela Giuliano, Antonietta Notaro, Sonia Emanuele

**Affiliations:** 1 Laboratory of Biochemistry, Department of Biological, Chemical and Pharmaceutical Sciences and Technologies (STEBICEF), University of Palermo, 90127 Palermo, Italy; federica.affranchi@unipa.it; 2 Department of Biomedicine, Neurosciences and Advanced Diagnostics (BIND), Biochemistry Building, University of Palermo, 90127 Palermo, Italy; giovanni.pratelli@unipa.it (G.P.); sonia.emanuele@unipa.it (S.E.); 3 Mater Società Consortile a.r.l., Via Brecce a S. Eramo 112/114, 80146 Naples, Italy; raffaeleraimondo@mater.it (R.R.); pavelkiselev@mater.it (P.K.)

**Keywords:** grape pomace, subcritical water extraction, polyphenols, antioxidant activity, cellular stress

## Abstract

In the context of the circular economy, the valorization of natural biomolecules from by-products has recently represented a major goal in health promotion. From this perspective, this study examined the antioxidant potential of Sicilian white grape pomace from the Pinot Gris variety, using subcritical water extraction as an eco-friendly and innovative method to recover bioactive compounds. Different extraction parameters allowed for comparing the potential of various fractions. Among these, the Subcritical Water Extract obtained after 5 min at 160 °C (SWE^160.1^) was rich in gallic acid and protocatechuic acid, as evidenced by characterization with UHPLC-Q Exactive Orbitrap-HRMS system. SWE^160.1^ showed efficacious antioxidant activity, as confirmed by DPPH assay and total polyphenol and flavonoid content. Interestingly, SWE^160.1^ displayed cytotoxic activity in tumor cell lines, while preserving the viability of non-tumor bronchial epithelial cells. Specifically, SWE^160.1^ protected these cells from exogenous oxidative stress, reducing the ROS levels and activating Nrf2-mediated antioxidant response. Surprisingly, upregulation of antioxidant enzymes (HO-1 and SOD-2) induced by SWE^160.1^ was maintained in the presence of lipopolysaccharide, indicating a specific involvement of SWE^160.1^ in the anti-inflammatory response. Finally, SWE^160.1^ was also able to limit the formation of stress granules following acute stress, thereby supporting its potential to maintain cellular homeostasis. Overall, this study highlights the potential of grape pomace as a source of active molecules to prevent oxidative stress and inflammation.

## 1. Introduction

Polyphenols are secondary metabolites synthesized by different parts of plants, where they perform structural functions or act for plant defense against microbial attacks and abiotic stress conditions [1]. These compounds are present in edible fruits and vegetables, grains, tea, coffee and chocolate [2]. Polyphenols represent a large family of more than 8000 different compounds, which can be classified depending on their chemical structure. Among the different classes, phenolic acids and flavonoids are well characterized [3].

A large number of studies have demonstrated the health potential of polyphenols. Due to their anti-inflammatory properties, they are able to counteract the risk of inflammatory chronic diseases [4]. Specifically, evidence has been provided that polyphenols inhibit pro-inflammatory enzymes, such as COX-2 (Cyclooxygenase-2) and iNOS (Inducible Nitric Oxide Synthase), and interleukin production, as well as influence macrophage functions [5,6].

The role exerted by polyphenols as regulators of cell redox balance appears more complex. Indeed, these molecules behave as pro-oxidants or antioxidants depending on their concentration or cell type [7]. In cancer cells, with imbalanced redox state, polyphenols act as pro-oxidants, increasing the cellular content of reactive oxygen species (ROS) and causing cell death [8]. For this reason, these compounds represent a potential coadjuvant strategy for cancer therapy.

Differently, several lines of evidence indicate that polyphenols act as potent antioxidants in non-tumor cells. This protective action is related to both ROS scavenger activity and ability to induce the production of antioxidant enzymes, including superoxide dismutase, catalase and glutathione peroxidase [9]. These effects are often mediated by the activation of Nrf2 (Nuclear factor erythroid 2-related factor 2), one of the most representative transcription factors involved in antioxidant response [10]. Therefore, by mitigating oxidative stress and the consequent cellular damage, polyphenols can contribute to cellular health and well-being. Consistent with these observations, it has been recently demonstrated that different polyphenols, including resveratrol, can mitigate aging, supporting the mitochondrial functions [11,12].

Polyphenols are also present in the waste of agricultural industries. In particular, grape pomace, which is the by-product of the wine-making process that contains pulp, stems, seeds and grape skins, is particularly rich in polyphenols and has displayed healthy properties [13,14]. Recently, Prata et al. have analyzed the composition, biological activities, and potential applications of the extracts from different varieties of white grape pomace (*Vitis vinifera* L.) [15]. The authors also provided evidence that wine pomace extracts show antioxidant potential, which is correlated with the ability to reduce ROS, as well as copper and iron ions, thus reducing the formation of free radicals. Moreover, grape pomace polyphenols have been shown to promote the cellular antioxidant enzymatic defenses [16].

Although evidence on the beneficial properties of grape pomace is consistent, it is important to consider that different conditions, including geographical origin, cultivation method, temperature, soil conditions, and grape variety, can profoundly influence the composition of grape pomace extracts, as well as the extraction method used [17]. One of the most common methods used to recover polyphenols was the extraction with a solvent, such as water, ethanol, or methanol [18,19]. Supercritical fluid extraction was introduced in the late 1980s. This extraction method appears to be more eco-friendly and useful, particularly for the extraction of essential oils, flavors, fragrances, and bioactive compounds from natural sources [20]. However, this method requires the addition of a co-solvent to enhance the extraction efficiency of polyphenols. Moreover, the experimental conditions must be constantly monitored, making the entire process more complex [21]. Successively, subcritical water extraction was introduced. This represents a greener technique that uses water at 50–200 °C and elevated pressure to extract bioactive compounds from plant materials without organic solvents. This method operates at lower pressures than supercritical fluid extraction, which reduces the energy use and safety risks, as well as offers high efficiency and selectivity for compounds including phenolics, flavonoids, and essential oils [22,23,24].

This study aimed to chemically characterize the extract obtained from white grape pomace of Vitis Vinifera, Pinot Gris variety, sourced from Sicilian crops and to evaluate its biological effects on tumor and non-tumor cell lines. In a previous paper, we showed the antiproliferative effect of a hydroalcoholic extract from the same matrix in two different cancer cell lines [25]. This effect was accompanied by the induction of oxidative stress and activation of autophagy. Here, we demonstrate that the subcritical water method is particularly efficient in the extraction of polyphenols. Intriguingly, while confirming antitumor activity of the extract in cancer cells, significant antioxidant and protective response was observed in a model of non-tumor cells.

## 2. Results

### 2.1. Characterization of the Fractions Obtained by Subcritical Water Extraction

Sicilian white grape pomace, the solid residue produced by local winemaking industries, was subjected to subcritical water extraction, a relatively new method for extracting less-polar compounds. This technique employs water in a subcritical state to extract organic components by influencing polarity changes. The extraction process resulted in collecting various subcritical water extract (SWE) fractions, as detailed in the Section 4.

First, the total content of phenols and flavones/flavanones was quantified using specific colorimetric methods. Table 1 shows the total polyphenol (TPC) and flavonoid (TFC) contents of the SWE fractions and their antioxidant potential measured by the DPPH radical-scavenging assay. The fractions obtained at the temperature of 140 °C showed the highest levels of total polyphenols. However, the major antioxidant activity was found in the extracts obtained at the highest temperature (160 °C), achieving a 50% reduction in DPPH radicals at the lowest concentrations.

Then, the different extracts were characterized by UHPLC-Q. This method is widely employed for the identification and characterization of bioactive compounds from different matrices and their respective by-products. The analysis identified the presence of various phytochemicals expressed as mg analyte per 100 g dry weight (DW) (Table 2). We found that the main phenolic acid components in all tested fractions were gallic acid and protocatechuic acid. It is noteworthy that the extracts obtained at 160 °C contained high concentrations of these two phenolic acids, reaching approximately 61 mg and 17 mg per 100 g DW, respectively. Moreover, these fractions showed a very low flavonoid content. Differently, the extracts obtained at lower temperatures were rich in flavonoids and flavonols; among the anthoxanthins, procyanidin B1, catechin, epicatechin and procyanidin B2 were the most abundant, especially in the extracts obtained at 140 °C.

### 2.2. Functional Properties of SWEs: Cytotoxicity Evaluation on Tumor and Non-Tumor Cells

**Figure 1:**
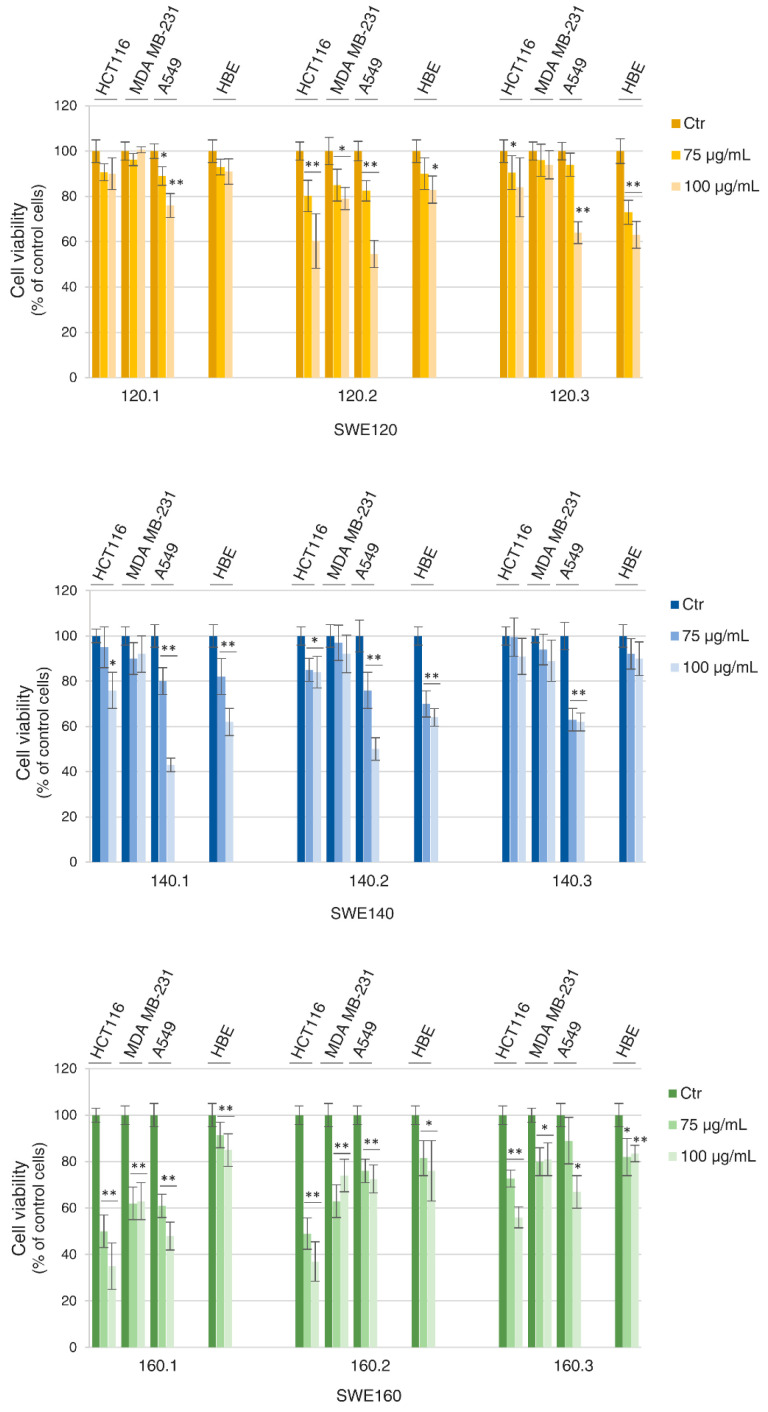
Dose-dependent effects of SWE fractions on the HCT116, MDA MB-231, A549 and HBE cell viabilities. Cell viability was assessed by MTT assay after 48 h of treatment. The SWE fractions were used at concentrations of 75 and 100 µg GAE/mL. The results are representative of three independent experiments and expressed as a mean ± SD. * *p* < 0.05, ** *p* < 0.01 vs. untreated control cells.

The different fractions of subcritical water extraction of Sicilian grape pomace were tested in tumor and non-tumor cell lines to evaluate their effects on the viability. Specifically, three different cancer cell lines—colon (HCT116), breast (MDA MB-231) and lung (A549) cancer cells—were used as tumor models, and HBE (Human Bronchial Epithelium cells) was used as a non-tumor model. Initially, dose-dependent effects of SWEs were assessed by MTT assay. As shown in Figure 1, some heterogeneous results were obtained with the different fractions of SWE obtained at 120 °C and 140 °C. Indeed, SWE^120.1^ and SWE^140.3^ did not affect the cell viability of either tumor or non-tumor cells. On the other hand, others, such as SWE^120.3^, SWE^140.1^, and SWE^140.2^, were found to be toxic in non-tumor HBE cells. In contrast, all the fractions obtained at the highest temperatures (160 °C) displayed significant toxicity in cancer cells, while being almost ineffective in HBE cells. When used at the highest concentration of polyphenols (100 µg GAE/mL), the SWE^160.1^ extract reduced the viability of HCT116 cells by approximately 70%, while exerting a lower effect on HBE cells, resulting in a reduction of about 18% (Figure 1).

**Figure 2:**
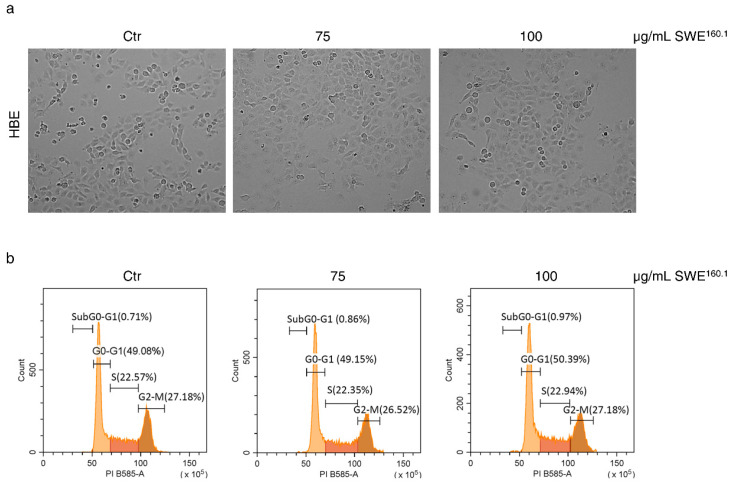
SWE^160.1^ treatment did not affect the HBE cell viability. (**a**) Representative images of HBE cells after 48 h of treatment with 75 and 100 µg GAE/mL SWE^160.1^. The cells were visualized under the microscope at 200× magnification, and the pictures were acquired by OptiKa Proview software, (version x64, 4.11.20805.20220506). (**b**) Flow cytometric analysis of the HBE cell cycle distribution after 48 h of 75 and 100 µg GAE/mL SWE^160.1^ treatment. The gates reported in the plots refer to the percentages of the cells in the different phases of cell cycle (G0-G1, S and G2-M). The SubG0-G1 gate indicates DNA-fragmented cells. The results are representative of two independent experiments.

Considering that we have previously characterised the antitumor effects of grape pomace extracts [25], the confirmed toxicity of SWE^160.1^ in cancer cells, together with the poor response of non-tumor cells, prompted us to specifically focus on HBE cells. Both morphological analysis and cell cycle distribution evaluation at 48 h treatment confirmed the non-toxic effect of SWE^160.1^ on HBE cells. Indeed, as reported in Figure 2, treatment with SWE^160.1^ produced no changes in the cell number and morphology (Figure 2a) and did not affect the cell cycle distribution (Figure 2b).

### 2.3. SWE^160.1^ Fraction Counteracted Reactive Oxygen Species (ROS) Production

**Figure 3:**
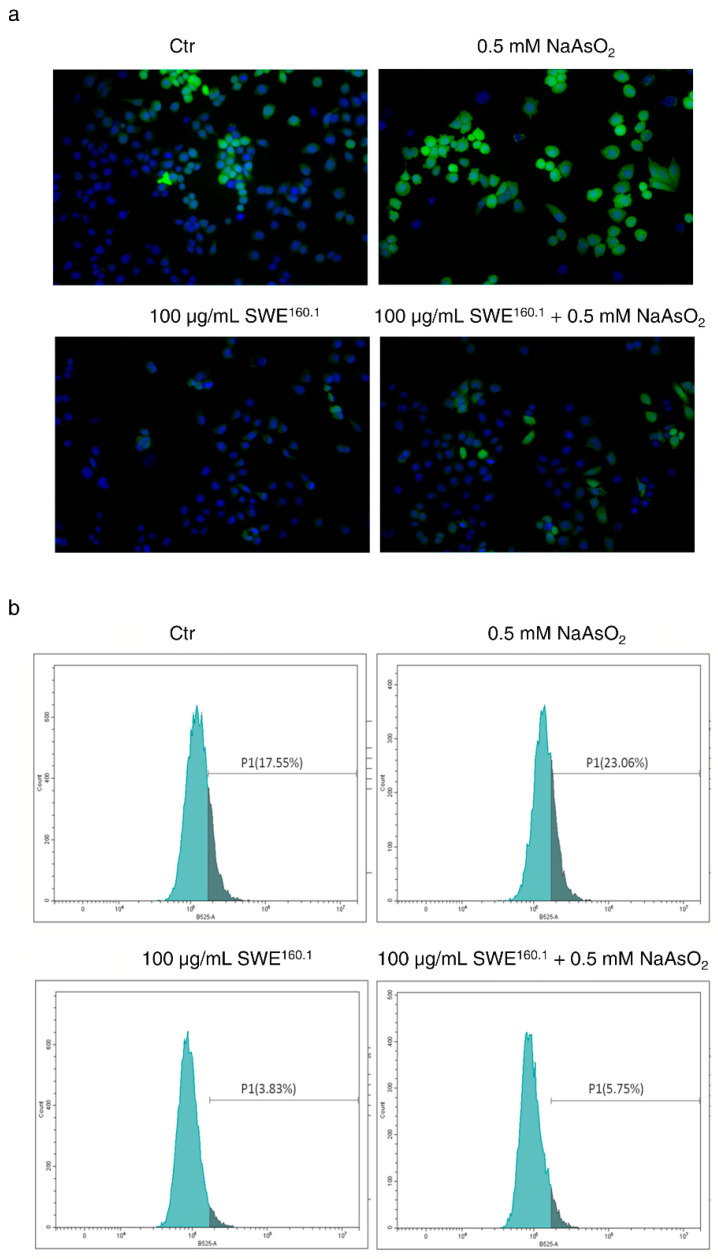
SWE^160.1^ counteracted the intracellular ROS level in basal and stress-induced conditions. (**a**) HBE cells were treated with 0.5 mM of NaAsO_2_ for 1 h or 100 µg GAE/mL SWE^160.1^ for 2 h alone or in addition to NaAsO_2_-treated cells. The presence of ROS after 2 µM H_2_DCFDA staining is shown by green fluorescence. Nuclei (blue) were stained with Hoechst 33342. The pictures were captured using a fluorescent microscope, acquired by OptiKa Proview software, at 200× magnification. (**b**) Flow cytometric analysis under the same conditions of treatment reported in (**a**) and stained with 0.5 mM H_2_DCFDA. The turquoise area represents a cell subpopulation with a low ROS intensity, whereas the grey area indicates a cell subpopulation with a high ROS intensity. The percentages reported in the plots refer to the grey area. The results are representative of two independent experiments.

Based on these preliminary results, we investigated whether the extract was able to exert an antioxidant action due to the high concentration of phenolic compounds. To this end, the effect of SWE^160.1^ treatment on ROS levels was first analyzed. As shown in Figure 3a, SWE^160.1^ reduced the green fluorescence of H_2_DCFDA ROS-sensitive probe under basal conditions, indicating a decrease in the cellular ROS level. Interestingly, SWE^160.1^ also significantly counteracted the production of ROS induced by sodium arsenite (NaAsO_2_), a potent oxidative stress inducer, used at a non-toxic concentration (0.5 mM).

Then, intracellular ROS levels were quantified using flow cytometry after staining with H_2_DCFDA (Figure 3b). The data shown in the plots indicate that SWE^160.1^ reduced the basal ROS levels by approximately 80%. Additionally, the extract decreased the ROS levels induced by NaAsO_2_ by about 75%, highlighting its potential to protect the cells from oxidative injury caused by external stressors.

**Figure 4:**
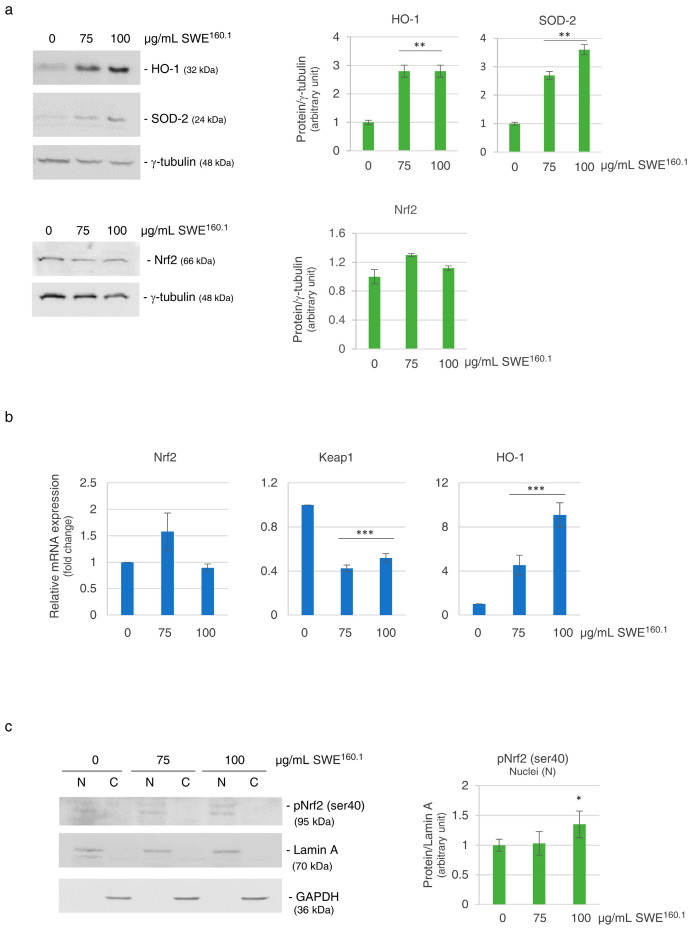
SWE^160.1^ treatment increased the antioxidant response. (**a**) Western blotting analysis of antioxidant factors (HO-1, SOD-2 and Nrf2) after 48 h of treatment with 75 and 100 µg GAE/mL SWE^160.1^ in HBE cells. Protein levels were normalized to γ-tubulin. (**b**) qRT-PCR analysis of Nrf2, Keap1 and HO-1 mRNA levels after 24 h of treatment with 75 and 100 µg GAE/mL SWE^160.1^ in HBE cells. Gene expression levels represent the relative mRNA expression compared to the untreated cells, normalized to GAPDH mRNA. (**c**) Western blotting of subcellular fractions of control and 48 h SWE^160.1^-treated cells incubated with pNrf2 (Ser40) antibody. Lamin A and GADPH antibodies marked as nuclei (N) and cytoplasmic (C) fractions, respectively. The images are representative of three different experiments. * *p* < 0.05, ** *p* < 0.01 vs. untreated control cells.

The observed reduction in ROS levels may be attributed to the induction of antioxidant enzymes. To verify this hypothesis, the levels of two key antioxidant enzymes, namely, HO-1 (heme oxygenase-1) and SOD-2 (superoxide-dismutase-2) [26,27], were assessed. After 48 h of treatment with 75 or 100 μg GAE/mL SWE^160.1^, both HO-1 and SOD-2 protein levels showed about a threefold increase (Figure 4a), suggesting that the extract upregulated cellular antioxidant systems. Quantitative RT-PCR confirmed that HO-1 mRNA increased more than eightfold in the presence of the highest concentration of the SWE (Figure 4b, right panel).

The cellular response to oxidative damage is often orchestrated by Keap1/Nrf2 axis. Nrf2 (nuclear factor erythroid 2-related factor 2) plays a central role in the antioxidant response, being the transcription factor responsible for regulating the expression of a wide range of protective antioxidant enzymes, including HO-1 and SOD-2 [28,29]. We found that SWE^160.1^ treatment did not significantly affect the expression of Nrf2 at both the protein and mRNA levels (Figure 4a, bottom panel, and Figure 4b, left panel).

However, since it is known that the activation of Nrf2 consists of its nuclear translocation, we evaluated the nuclear localization of the phosphorylated active form of Nrf2 (pNrf2). Interestingly, pNrf2 was found significantly increased in the nuclear fraction of treated cells (Figure 4c), supporting its role in the antioxidant response.

The nuclear translocation of Nrf2 is tightly controlled by Keap1 (Kelch-like ECH-associated protein 1), a component of E3 ubiquitin ligase complex, which is involved in the ubiquitination and proteasome degradation of Nrf2 under homeostatic conditions [28]. Interestingly, RT-PCR data demonstrated that after 24 h of treatment, the expression of Keap-1 was downregulated by approximately 50% in the treated HBE cells (Figure 4b, middle panel).

**Figure 5:**
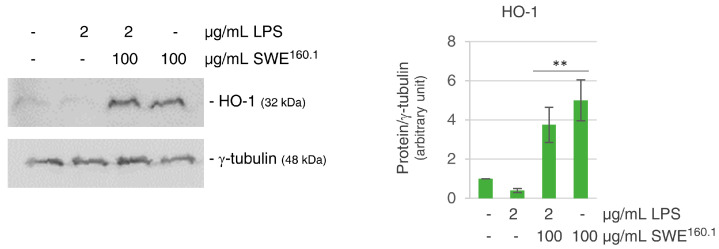
SWE^160.1^ treatment overwhelmed LPS-induced HO-1-reduction. Western blotting analysis of HO-1 enzyme after 48 h of treatment with 2 µg/mL LPS alone or in combination with 100 µg GAE/mL SWE^160.1^. Protein levels were normalized to γ-tubulin. Densitometric analysis, performed using Quantity One software, (version 4.6.6), is shown in the histogram. The result is representative of two independent experiments, with values expressed as mean ± SD. ** *p* < 0.01 vs. untreated control cells.

It was also interesting to evaluate whether SWE-dependent HO-1 increase could contribute to the cellular response under inflammatory conditions. HO-1 is typically upregulated in response to LPS (lipopolysaccharide)-induced inflammation due to its crucial roles in counteracting not only oxidative stress but also inflammatory damage [30]. In contrast, in our study, LPS exposure resulted in an approximately 50% decrease of HO-1 levels compared with unstimulated cells. However, the presence of SWE^160.1^ effectively overwhelmed the LPS-induced downregulation, maintaining the levels of HO-1 comparable with those observed in the cells treated with SWE^160.1^ alone (Figure 5).

### 2.4. SWE^160.1^ Prevented the Formation of Stress Granules Induced by an Acute Insult

Oxidative stress is a condition that also triggers the formation of membrane-less stress granules (SGs), which are transient cytoplasmic aggregates of large messenger ribonucleoprotein (mRNP) complexes [31]. They function as storage compartments contributing to the stress-induced suppression of global protein synthesis. Moreover, the granules formation is influenced by different types of oxidative stress playing a crucial role in regulating redox homeostasis.

**Figure 6:**
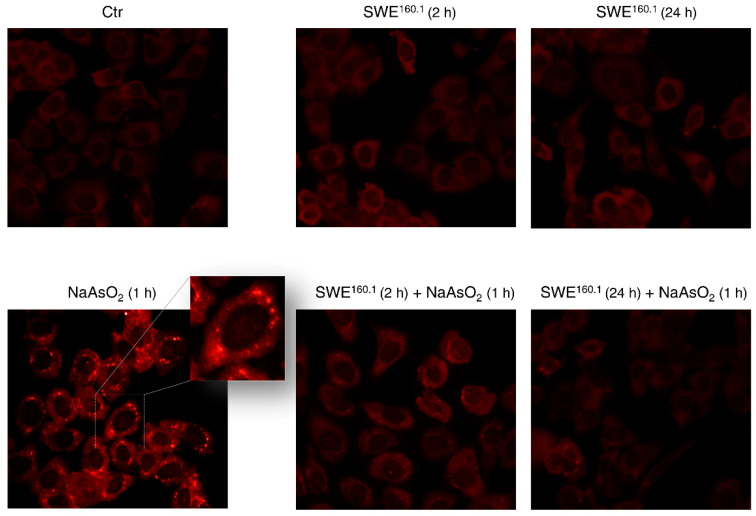
SWE^160.1^ treatment prevented sodium-arsenite-induced stress granules. Representative images of immunofluorescence experiments using an antibody against G3BP1 protein. Cells were treated with 100 µg GAE/mL SWE^160.1^ for 2 or 24 h alone or followed by 1 h treatment with 0.5 mM NaAsO_2_. Cells treated for 1 h with 0.5 mM NaAsO_2_ were considered as a positive control for stress granule induction. Cells were visualized under a fluorescent microscope at 200× magnification, and images were acquired by OptiKa Proview software. The pictures are representative of two independent experiments. The insert shows an enlarged view of a representative detail.

Using immunofluorescence microscopy, we investigated whether HBE cells could form SGs under stress conditions induced by sodium arsenite exposure. For this purpose, cells were cultured with 0.5 mM NaAsO_2_ to induce stress granule formation. After 1 h of sodium arsenite treatment, cells were stained using an antibody against G3BP1 (Ras GTPase-activating protein-binding protein 1), an SG marker. As shown in Figure 6, NaAsO_2_ induced the appearance of membrane-less granules visible as bright red spots in the pictures, while SWE^160.1^ alone did not induce the appearance of stress granules being red fluorescence diffused throughout the cells, similarly to that observed in control cells. Notably, brief (2 h) or prolonged (24 h) pre-treatment with SWE^160.1^ before NaAsO_2_ exposure prevented the formation of granules induced by the stressor. 

## 3. Discussion

This study represents the first evidence of the redox-modulating capability of a subcritical water extract (SWE) from white grape pomace of Vitis vinifera cultivated in western Sicily. Using the innovative and eco-friendly subcritical water extraction method resulted in an extract richer in polyphenols compared with the one obtained by hydro-alcoholic extraction from the same matrix [25]. This also implied less time and milder experimental conditions. The characterization of the extract obtained in the first 5 min at 160 °C (SWE^160.1^) by a UHPLC-Q system revealed enrichment in gallic acid and protocatechuic acid at concentrations of 61.9 mg/100 g and 17.6 mg/100 g dry weight, respectively. Differently, when the same matrix was subjected to a hydro-alcoholic extraction protocol the concentrations of the same phenolic acids resulted significantly lower (20.96 mg/100 g dry weight for gallic acid and 4.47 mg/100 g dry weight for protocatechuic acid) [25]. This is in accordance with the findings present in the literature that emphasize the advantages of the subcritical water method to extracting bioactive compounds from natural matrices [23,32].

However, it must be considered that the analysis of the biological effects of the different fractions obtained by subcritical water extraction on various cell lines (tumor and non-tumor) revealed diversified effects. This could be related to the heterogeneous composition of the extracts. Indeed, although the matrix was the same, the extraction parameters led to the recovery of different quality and quantity of bioactive molecules. The fractions obtained at 120 and 140 °C exhibited generalized toxicity or, conversely, no cytotoxicity at all. Chemical analysis of these fractions revealed a higher flavonoid content than the extracts obtained at 160 °C. Although many reports show that flavonoids protect various cell types from oxidative stress, high concentrations may be toxic to normal human cells [33].

It is important to keep in mind the hormetic role of this class of phytocompounds. Many polyphenols, acclaimed for their protective and beneficial properties in normal cells, show cytotoxic effects at higher concentrations. In this regard, Calabrese et al. demonstrated the hormetic dose/response of quercetin and concluded that this role is independent of the biological model or cell type [34]. Moreover, since we used crude extracts, it is also possible to hypothesize that the observed effects are due to additive effects of the individual components present at different concentrations.

Regarding the different effects the extract has on tumor and non-tumor cells, these could be related to the high basal ROS content in tumor cells, which most likely undergo oxidative stress more easily than normal cells under the effects of polyphenols. Conversely, in non-tumor HBE cells, the SWE^160.1^ extract promoted a significant antioxidant effect, significantly reducing the intracellular ROS level and counteracting the effects of sodium arsenite, a potent oxidative stressor. HBE cells were specifically selected as a proper non-tumor model considering that the respiratory epithelium is continuously exposed to endogenous and exogenous sources of oxidative stress. Therefore, HBE cells represent a physiologically relevant system to evaluate the antioxidant activity of the grape pomace extract.

Studying the effect of SWE^160.1^ over time prompted us to hypothesize a double-action mechanism that is probably time-dependent. The rapid reduction in ROS levels observed after 2 h of treatment may be attributed to a direct antioxidant and ROS-scavenging activity of the polyphenol-rich extract. Indeed, polyphenols are known to neutralize reactive oxygen species through electron- or hydrogen-donating mechanisms and, in some cases, by chelating redox-active metal ions involved in ROS generation [35]. This early effect is consistent with the ability of the low molecular weight polyphenolic compounds, such as gallic acid, to rapidly enter the cells through passive diffusion across the plasma membrane and/or transporter-mediated uptake [35,36] (https://doi.org/10.3390/antiox9121263) (accessed on 1 May 2026). On the other hand, we cannot exclude the hypothesis that polyphenols inhibit the enzymes responsible for the production of ROS through the interaction with transient receptor potential channels [37].

In contrast, the modulation of antioxidant enzymes observed after longer exposure likely reflects a later adaptive cellular response involving transcriptional activation of antioxidant defense pathways rather than an immediate scavenging effect. Indeed, after 48 h of SWE exposure, we observed a significant upregulation of HO-1 and SOD-2 levels, two enzymes belonging to the Nrf2 pathway [38]. This highlighted the ability of the extract to enhance cellular redox resilience by regulating Keap1/Nfr2 pathway. Keap1 (Kelch-like ECH-related protein 1) functions as an inhibitory protein of Nrf2 activity [39,40].

The protective role of SWE^160.1^ is not limited to the antioxidant response. Sometimes, cells under starvation or other different cell stresses form dynamic non-membranous cytoplasmic aggregates, named stress granules, to modulate protein synthesis by sequestering mRNAs and riboproteins [41,42]. Although initially associated with neurological diseases, recent studies have demonstrated the ability of different inducers, including natural compounds, to either stimulate or inhibit the formation of stress granules, opening new therapeutic perspectives [43]. Quercetin, for instance, has been shown to promote stress granules disassembly via G3BP1 modulation in astrocytes exposed to HIV-1 gp120 [44] while also regulating stress response mechanisms, such as eIF2α phosphorylation, to reduce protein aggregation and amyloid β production in Alzheimer’s models [45]. Moreover, curcumin, resveratrol, and various ginsenosides influence stress-signaling cascades, protein aggregation equilibria, and translation initiation, which each represent a critical node in stress granule regulation [46]. In accordance, the findings reported in this study highlight SWE^160.1^ ability to preserve cellular homeostasis of HBE cells at multiple levels, also inhibiting stress granules formation upon exposure to sodium arsenite, which is one of the most effective SG promoters compared with other stress inducers [47]. Unlike many other well-known stress granules inhibitors, such as cycloheximide, which causes global translational inhibition and exhibits high toxicity [48], SWE did not induce cellular toxicity or growth defects, as demonstrated by an MTT assay and evaluation of cell cycle distribution. Since stress granules are associated with a wide range of pathological conditions, including neurodegenerative disorders and cancer [49], preventing their assembly or promoting the disassembly of aberrant granules may serve as valuable tools in disease models onset, thereby facilitating a deeper understanding of the contribution of these aggregates to disease development and progression.

Finally, the slight decrease in HO-1 level after the treatment of HBE cells with lipopolysaccharide is reported in this paper and deserves further investigation. This result is in contrast with the findings reported in the literature [30,50]. Indeed, it is well known that LPS triggers Nrf2 nuclear translocation with the consequent activation of HO-1 expression, which reduces NF-kB activation and the inflammation pathway [51]. Similar results were observed in acute lung injury models in epithelial cells [52]. Differently, our result is in accordance with the study of Peng et al. [53], which demonstrated that certain conditions lead to reduction or dysregulation of HO-1 expression as a result of inflammatory signaling pathway activation. Moreover, LPS can significantly inhibit the expression of the Nrf2 signaling pathway in THP-1 cells [54]. Intriguingly, when grape pomace extract was added to LPS-treated cells, the level of HO-1 was restored, indicating a regulatory effect. The molecular basis of this atypical behavior will be investigated. Here, in accordance with Tonolo et al. [55], we speculate that SWE could inactivate inflammation proteins by inducing the Nrf2 signaling pathway. Therefore, SWE160.1 may help restore the protective function of HO-1, supporting its potential therapeutic relevance beyond its antioxidant activity, particularly in the broader context of inflammatory diseases.

## 4. Materials and Methods

### 4.1. White Grape Pomace Recovery and Extraction

White grape pomace was supplied by the Testa-Ferrarella’s agri-food industry (Trapani, Sicily) from the 2023 and 2024 harvests of the Vitis vinifera cultivar “Pinot Gris”. The raw material, consisting of grape skins, seeds, and stems, was sun-dried by the industry and stored at −80 °C until required. Before the extraction, the crude material was ground into a fine powder.

The subcritical water extraction was conducted in a bench-top high-pressure system provided by Extratex SFI (France). For each experiment, 180 g of white grape pomace powder was loaded into the extractor vessel. The working pressure was kept constant at 100 bar to ensure the water stayed in the liquid phase at all the tested temperatures (120, 140, and 160 °C). The water flow rate (20 g/min) and the total extraction time (15 min) were kept constant throughout all extractions. The aqueous extracts were collected in three distinct sequential fractions 1, 2 and 3 (0–5, 5–10 and 10–15 min, respectively) using separate containers. These fractions were labelled as SWE (Subcritical Water Extract)^temperature (°C).1^, SWE^temperature (°C).2^, and SWE^temperature (°C).3^, respectively. Instantly after the collection, the extracts were centrifuged at 3880 *g* for 15 min, and then at 15,500 *g* for 20 min at 4 °C. Finally, the extracts were filtered through a 0.22 μm filter and stored at −20 °C until further use. The dry weights of 1 mL of each extract were calculated once lyophilized and are reported in the Table 3 below.

### 4.2. Colorimetric Assays of Total Polyphenol and Flavonoid Content

The total polyphenol concentration in the different fractions was determined using the Folin–Ciocalteu colorimetric reagent, which reacts with the phenolic compounds. The samples were prepared as previously reported [25]. The concentration of the extracts used in the experiments was based on their polyphenol content and expressed as μg of gallic acid equivalent (GAE)/mL since gallic acid was used for the calibration curve [56].

The total flavonoid content was determined, according to Attard et al. [57], with some modifications [58]. For the assay, the extract was mixed with a solution of aluminum chloride (AlCl_3_) 0.3% (*w*/*v*) for 1 min. A total of 200 μL of 1 mM NaOH was added, and 10 min later, the absorbance was measured spectrophotometrically at 410 nm. The total flavonoid content was expressed in μg quercetin equivalent (QE)/mL since quercetin was used for the calibration curve.

### 4.3. Evaluation of Antioxidant Activity by DPPH (2,2-Diphenyl-1-Picrylhydrazyl)

DPPH radical scavenger activity of grape pomace extracts was determined according to Attard et al. [54]. A stock solution of DPPH (60 μM) was prepared fresh in methanol and maintained at 4 °C in the dark. Subcritical water extracts (50 μL) were added to each well and serially diluted two-fold in a 96-well microplate to obtain decreasing concentrations down to the lowest tested level. Then, 150 μL of DPPH solution was added to each well, and the reaction was incubated in the dark for 30 min at room temperature. Both DPPH alone and the extract alone were employed as negative controls. The absorbance was measured at 540 nm using a microplate reader (OPSYS MR, Dynex Technologies, Chantilly, VA, USA).

The DPPH free radical scavenging activity, expressed as IC50 (50% inhibitory concentration), was calculated by the following equation:

$$\%\;\mathrm{inhibition}=\frac{\left[\left({\mathrm{Abs}}_{\mathrm{Blank}}{-\mathrm{Abs}}_{\mathrm{control}}\right){-\mathrm{Abs}}_{\mathrm{sample}}\right]}{{\mathrm{Abs}}_{\mathrm{Blank}}{-\mathrm{Abs}}_{\mathrm{control}}}\times 100$$

### 4.4. Qualitative and Semi-Quantitative Analysis of the Extracts

UHPLC-Q Exactive Orbitrap-HRMS system (Thermo Fisher Scientific™, Bremen, Germany) composed of a Dionex Ultimate 3000 liquid chromatograph coupled to a Q Exactive™ Plus Hybrid Quadrupole-Orbitrap™ Mass Spectrometer equipped with a heated electrospray ionization (HESI) ion source (Headquarters, Waltham, MA, USA) was employed to evaluate the polyphenol compounds of SWEs.

Chromatographic separation was achieved on Luna C18(2) (150 × 2.0 mm, 5 µm) equipped with a precolumn, with 0.1% formic acid in water (mobile phase A) and 0.1% formic acid in methanol (mobile phase B). A gradient method at 200 μL/min flow rate was applied as follows: started at 5% B, stayed for 2 min; increased to 95% B over 18 min, held for 2 min; then decreased to 5% B over 18 min; and maintained constant for 2 min for a total run time of 40 min. The injection volume was 1 μL. Full mass and targeted SIM (t-SIM) scan methods were applied. The Orbitrap parameters were set as follows: negative (−) ESI full scan mode and t-SIM, sheath gas flow rate 30 AU, discharge voltage 3.0 kV, capillary temperature 300 °C, resolution 35,000 FWHM, AGC target 5 × 10^6^, maximum injection time 200 ms, and scan range 80–1000 *m*/*z*.

Calibration curves were constructed at five calibration levels for p-hydroxybenzoic acid, protocatechuic acid, p-coumaric acid, gallic acid, kaempferol, and quercetin. When reference compounds were not available, the calibration of structurally related substances was used.

### 4.5. Cell Cultures, Treatment Conditions and Reagents

Human Bronchial Epithelial (16-HBE) cell line was purchased from Merck Millipore (Merck Life Science, Milan, Italy); HCT116, MDA MB231 and A549 cells were provided by the Interlab Cell Line Collection (ICLC, National Institute of Cancer Research, Genoa, Italy). Cells were maintained in Dulbecco’s Modified Eagle’s Medium (DMEM) containing 2 mM glutamine, 10% heat-inactivated fetal bovine serum (FBS), 100 U/mL penicillin and 100 μg/mL streptomycin. Cultures were maintained in an incubator at 37 °C in a humidified environment with 5% CO_2_.

For the experiments, cells were seeded in the appropriate culture plate. Following overnight incubation, cells were treated with 75 or 100 μg GAE/mL SWE^160.1^ for the designated times. Control cells were incubated with the vehicle alone. All reagents were purchased from Merck (Milan, Italy), unless otherwise stated.

### 4.6. Cell Viability Assay by MTT

The colorimetric MTT assay (3-(4,5-dimethylthiazol-2-yl)-2,5-diphenyltetrazolium bromide) was used to evaluate cellular metabolic activity as an indicator of cell viability and proliferation. The cells were seeded in 96-well plates in 200 μL of culture medium, and test agents were added after overnight incubation. After the treatment for 48 h with SWE^160.1^, MTT was performed as previously reported [59]. The absorbance was measured at 570 nm by using a microplate reader (OPSYS MR, Dynex Technologies, Chantilly, VA, USA), and the viability of the treated cells was expressed as a percentage of the optical density (OD) values relative to the untreated, control cells.

### 4.7. Cell Cycle Analysis

To evaluate the cell cycle distribution, after 48 h SWE^160.1^ treatment, the cells were collected by trypsinization (0.025% trypsin-EDTA; Life Technologies Ltd., Monza, Italy) and resuspended in a hypotonic solution containing 25 µg/mL propidium iodide, 0.1% sodium citrate, 0.4% Igepal, and 10 µg/mL RNase A. The distribution of cell cycle phases was assessed using a Cytoflex Flow Cytometer (Beckman Coulter Life Sciences, Indianapolis, IN, USA) and CytExpert 2.5 software. Cell debris and aggregates were excluded by setting an appropriate gating. At least 30,000 events per sample were considered, with data recorded in list mode files. The results shown in the figures are representative of two independent experiments, each performed with duplicated samples.

### 4.8. Analysis of Reactive Oxygen Species (ROS)

Reactive oxygen species (ROS) levels were assessed using fluorescence microscopy and flow cytometry in the presence of the oxidation-sensitive dye 2′,7′-dichlorodihydrofluorescein diacetate (H_2_DCFDA) (Molecular Probe, Life Technologies, Eugene, OR, USA).

For the microscopy analysis, cells were treated with NaAsO_2_ (0.5 mM) for 1 h or 100 µg GAE/mL SWE^160.1^ for 2 h alone or in addition to NaAsO_2_-pretreated cells in 24-well plates. Then, the cells were washed with PBS and incubated with 2 μM H_2_DCFDA dye for 15 min in the dark at 37 °C and 5% CO_2_. Simultaneously, the cells were stained with 2.5 μg/mL Hoechst 33342 (H3570, Invitrogen by Thermo Fisher Scientific™, Bremen, Germany) to visualize the nuclei under the same conditions. After incubation, excess dye was removed, and the cells were washed with PBS. Fluorescence was examined using a fluorescence microscope: the signal from 2′,7′-dichlorofluorescein (DCF), indicating intracellular oxidation, was captured using a FITC filter (488 nm excitation, 530 nm emission), while the Hoechst 33342 signal was observed using a DAPI filter (372 nm excitation, 456 nm emission). Images were captured at 200× magnification using an Optika IM3FL4 fluorescence microscope with a computer imaging system (OPTIKA PROVIEW, version x64, 4.11.20805.20220506).

For the flow cytometry analysis, after treatment in 6-well plates, the cells were washed, trypsinized, and centrifuged at 100 *g*. They were then stained with 0.5 μM H_2_DCFDA for 50 min in pre-warmed phenol red-free medium. After incubation, the cells were promptly examined using a Cytoflex Flow Cytometer (Beckman Coulter Life Sciences, Indianapolis, IN, USA) with CytExpert software.

### 4.9. Western Blotting Analysis and Antibodies

Western blotting was conducted to evaluate the expression levels of proteins associated with the activated pathways. After 48 h of treatment with the extract (75 or 100 μg GAE/mL), cells were detached and lysed as previously reported [25]. Protein concentration was quantified using the Bradford Protein Assay (Bio-Rad Laboratories Srl., Milan, Italy), and 30 μg of each protein sample was loaded onto a polyacrylamide gel for electrophoresis under denaturing conditions. After electroblotting, the nitrocellulose membranes were incubated with specific primary antibodies targeting the proteins of interest, following the datasheets information. Anti-HO-1 (GTX101147) was sourced from Gene Tex (GeneTex, Prodotti Gianni, MI, Italy); anti-SOD-2 (sc-133) and anti-Nrf2 (Sc-722) from Santa Cruz Biotechnology (Heidelberg, Germany); anti-pNrf2 (Ser40) (SAB5701902), anti-Lamin A (MABT330) and anti-γ-tubulin (T9026) from Merck Millipore (Merck Life Science, Milan, Italy); anti-G3BP1 (G6046) from Cell Signaling Technology (Danvers, MA, USA); and anti-GAPDH (Glyceraldehyde-3-Phosphate Dehydrogenase) (AM4300) from Thermo Fisher Scientific™ (Bremen, Germany). γ-tubulin was employed to normalize the Western blotting signals. HRP-conjugated secondary antibodies were purchased from Promega (Milano, Italy).

### 4.10. Subcellular Fractionation

Nuclei and cytoplasm fractions were prepared from cells at approximately 80% confluence after 48 h SWE^160.1^ treatment. Briefly, the cells were resuspended in RBS100 buffer (10 mM Tris-HCl, pH 7.4, 100 mM NaCl, 2.5 mM MgCl_2_) containing 0.1% Igepal and a mixture of protease inhibitors, incubated for 10 min at 4 °C. Cells were lysed by passing through a 25-gauge needle 10 times on ice. The suspension was centrifuged at 900 *g* for 10 min at 4 °C to obtain a pellet (nuclei) and a supernatant (cytoplasmic fraction). Nuclei were resuspended in RSB 100 buffer and sonicated. Both nuclei and cytoplasmic fractions were quantified using the Bradford protein assay and analyzed through Western blotting. Lamin A and GAPDH antibodies were used as cellular fractionation controls.

### 4.11. Quantitative Real Time-PCR Analysis

Cells were harvested using an appropriate volume of QIAzol Lysis Reagent (Qiagen). Total RNA was extracted from 1 × 10^6^ cells treated with 75 and 100 µg GAE/mL SWE^160.1^ for 24 h using a Direct-zol RNA MiniPrep kit (Zymo Research), following the manufacturer’s instructions. The integrity and purity of the RNA extracted were assessed by agarose gel electrophoresis.

cDNA was obtained from 1 μg of RNA using the Maxima First Strand cDNA synthesis kit (Thermo Fisher Scientific™, Bremen, Germany) and was subsequently diluted (4×) with PCR-grade water. qRT-PCR was performed with SYBR Green SuperMix (Bio-Rad) in 96-well plates according to the manufacturer’s instructions using IQ5 Cycler instrument (Bio-Rad). qPCR was performed in triplicate and repeated for confirmation. Data processing and statistical analysis were performed using IQ5 cycler software, 2.0 version. The relative quantification in gene expression was determined using the 2^−ΔΔCt^ method.

Reactions excluding the reverse transcriptase enzyme were used as negative control. GAPDH was employed as a housekeeping gene. The primers used were GAPDH (Proligo USA, Milan, Italy), Nrf2 (HP209154, OriGene Technologies, Inc., Rockville, MD, USA) and HO-1 (HP205872, OriGene Technologies, Inc., USA). Primer sequences and thermocycling conditions used for the amplification are reported in Table 4 and Table 5.

### 4.12. Immunofluorescence Assay

For immunofluorescence experiments, cells were treated with 100 µg GAE/mL SWE^160.1^ for 2 or 24 h alone or followed by 1 h treatment with 0.5 mM NaAsO_2_ on a glass coverslip. Then the cells were fixed with 4% paraformaldehyde (*v*/*v*) for 20 min and incubated overnight at 4 °C for G3BP1 detection, as previously described [60]. Cells were then incubated for 1 h with the secondary Cy3-conjugated donkey anti-rabbit antibody at 1:1000 dilution (AP182C, Merck-Sigma Aldrich, Milan, Italy). Images were captured at 200× magnification using the Cy3 filter (554 nm excitation, 568 nm emission) of the Optika IM3FL4 fluorescence microscope with a computer imaging system (OPTIKA PROVIEW, version x64, 4.11.20805.20220506).

### 4.13. Statistical Analysis

Data are presented as the mean ± S.D., and statistical analyses were performed using the Student’s *t*-test and one-way analysis of variance (ANOVA). Comparisons were made between the control (untreated) group and all treated samples. Statistical significance was determined using one-way ANOVA, followed by Bonferroni’s post hoc test. *p*-values of less than 0.05 were considered statistically significant.

## 5. Conclusions

In the present research, using the subcritical water extraction process, we characterized an extract from grape pomace rich in polyphenols, with potent antioxidant properties in human bronchial epithelial cells. Molecular investigations indicated that the extract reduced ROS levels and triggered an antioxidant cell response. Moreover, it also limited the formation of stress granules and seemed to play a role in contrasting the inflammation. Overall, this study reinforces the hypothesis of a potential use as a supplement in functional foods and in nutraceutical/pharmaceutical formulations.

## Figures and Tables

**Table 1:** Evaluation of polyphenols and flavonoids contents and antioxidant potential of the different SWE fractions.

Sample	TPC (µg GAE/μL)	TFC (µg QE/μL)	DPPH (mg DW/mL)
SWE^120.1^	2.275 ± 0.31	0.85 ± 0.007	5.12 ± 0.2
SWE^120.2^	4.175 ± 0.38	2.05 ± 0.29	2.2 ± 0.09
SWE^120.3^	2.4 ± 0	1.12 ± 0.04	24.4 ± 0.8
SWE^140.1^	4.31 ± 0.39	1.47 ± 0.17	9.4 ± 0.1
SWE^140.2^	3.92 ± 0.15	1.65 ± 0.01	1.44 ± 0.3
SWE^140.3^	2.87 ± 0.31	1.4 ± 0.03	1.31 ± 0.27
SWE^160.1^	2.29 ± 0.06	1.2 ± 0.38	5.5 ± 0.38
SWE^160.2^	2.17 ± 0.38	1.17 ± 0.40	4.1 ± 0.41
SWE^160.3^	1.6 ± 0.17	0.8 ±0.31	1 ± 0.3

The concentrations of polyphenols (TPC), expressed as µg of gallic acid equivalents (GAE)/µL; flavonoids (TFC), expressed as µg of quercetin equivalents (QE)/µL; and antioxidant potentials, expressed as mg of dry weight (DW)/mL, present in the different extracts ± S.D. are shown.

**Table 2:** Individual components identified in the different SWE fractions.

Phenolic Acid (mg/100 g Dry Weight)									
	SWE 120 °C			SWE 140 °C			SWE 160 °C		
Analytes	1	2	3	1	2	3	1	2	3
Gallic acid	16.125	27.86	27.325	41.893	39.211	48.193	61.965	56.423	52.972
Protocatechuic acid	3.7	7.194	6.369	10.1	10.842	14	17.642	15.71	13.981
Caffeic acid	0.145	0.968	0.425	1.173	1.1684	2.3935	0.4809	0.5233	0.6572
Caftaric acid	2.875	3.097	2.2875	14.136	10.336	8.9477	NF	NF	NF
Syringic acid	NF	NF	NF	NF	NF	NF	5.6393	4.491	3.5258
p-Coumaric acid	0.065	0.3889	0.1875	0.51	0.5	0.5419	NF	NF	NF
Ferulic acid	NF	NF	NF	2.9466	2.4789	2.9355	NF	NF	NF
Fertaric acid	1.055	0.4757	0.45	1.8466	NFR	NF	NF	NF	NF
p-hydroxybenzoic acid	0.235	0.4931	0.4313	1.243	1.426	1.561	0.5865	0.5304	0.4977
**Anthoxanthins (Flavonoids and Flavonols) (mg/100 g Dry Weight)**									
Procyanidin B1	3.9	6.691	5.925	17.326	19.663	25.793	NF	NF	NF
Catechin	13	19.92	20.525	45.783	67.131	84.445	0.3988	0.362	0.352
Epicatechin gallate	0.0425	2.2118	0.525	4.9	4.3842	7.3225	0.0792	NF	NF
Procyanidin B2	2.35	4.25	3.5125	4.49	5.321	6.4709	NF	NF	NF
Epicatechin	7.865	13.056	12.913	31.373	42.256	49.45	0.2317	0.2043	0.174
Quercetin 3-O-glucoside	0.41	0.836	0.587	3.68	3.69	4.167	0.076	NF	NF
Quercetin 3-O-glucuronide	0.585	0.705	0.5875	6.4866	6.936	7.258	NF	NF	NF
Quercetin 3-O-galactoside	NF	NF	NF	0.1033	0.1	0.14	NF	NF	NF
Quercetin 3-O-rhamnoside	NF	NF	NF	NF	NF	NF	NF	NF	NF
Kaempferol 3-O-glucoside	NF	NF	NF	0.78	0.6579	0.7742	NF	NF	NF
Quercetin	NF	NF	NF	11.423	11.781	18.193	0.9765	0.7706	0.366
Kaempferol	NF	NF	NF	0.4433	0.4262	0.7677	0.0264	0.0215	NF

Data are reported as mg/100 g dry weight. NF, not found.

**Table 3:** Dry weight (DW) of the different fractions of SWE.

Extracts	Dry Weight (DW)
SWE^120.1^	20 mg/mL
SWE^120.2^	28.8 mg/mL
SWE^120.3^	16 mg/mL
SWE^140.1^	30 mg/mL
SWE^140.2^	19 mg/mL
SWE^140.3^	15.5 mg/mL
SWE^160.1^	34.1 mg/mL
SWE^160.2^	27.9 mg/mL
SWE^160.3^	21.3 mg/mL

SWE fractions were obtained using different temperatures (120, 140, and 160 °C) and recovered after different extraction times (SWE^temperature (°C).1 (0−5 min)^, SWE^temperature (°C).2 (5−10 min)^, SWE^temperature (°C).3 (10−15 min)^).

**Table 4:** Primer sequences.

Primer	Forward	Reverse
GAPDH	TGACATCAAGAAGGTGA	TCCACCACCCTGTTGCTGTA
Nrf2	CACATCCAGTCAGAAACCAGTGG	GGAATGTCTGCGCCAAAAGCTG
HO-1	CCAGGCAGAGAATGCTGAGTTC	AAGACTGGGCTCTCCTTGTTGC
Keap1	CAACTTCGCTGAGCAGATTGGC	TGATGAGGGTCACCAGTTGGCA

**Table 5:** Thermal cycling conditions.

Primer	Thermal Profile
Nrf2, HO-1 and Keap1	95 °C, 10 min, 1 cycle. 95 °C, 30 s, 60 °C, 60 s, 72 °C, 30 s and 72 °C, 5 min, 40 cycles.
GAPDH	95 °C, 10 min 1 cycle. 95 °C, 30 s, 60 °C, 60 s, 72 °C, 30 s and 72 °C, 5 min, 30 cycles.

## Data Availability

The data that support the findings of this study are available from the corresponding author upon reasonable request.
